# Successful resection of a slow-growing synchronous pulmonary metastasis from distal cholangiocarcinoma resected 3.5 years after initial surgery: a case report

**DOI:** 10.1186/s13256-018-1671-6

**Published:** 2018-05-22

**Authors:** Teruhisa Sakamoto, Soichiro Honjo, Masaki Morimoto, Masataka Amisaki, Yosuke Arai, Naruo Tokuyasu, Keigo Ashida, Hiroaki Saito, Kanae Nosaka, Yoshiyuki Fujiwara

**Affiliations:** 1 Division of Surgical Oncology, Department of Surgery, Faculty of Medicine, Tottori University, 36-1 Nishi-cho, Yonago, 683-8504 Japan; 2 Division of Organ Pathology, Department of Pathology, Faculty of Medicine, Tottori University, Yonago, 683-8503 Japan

**Keywords:** Synchronous pulmonary metastasis, Distal cholangiocarcinoma, Lepidic growth

## Abstract

**Background:**

A few reports have described the effectiveness of resection for recurrent cholangiocarcinoma. However, none have described resection of synchronous pulmonary metastasis from distal cholangiocarcinoma. We report the first case, to the best of our knowledge, of a slow-growing synchronous pulmonary metastasis from distal cholangiocarcinoma resected 3.5 years after the initial surgery.

**Case presentation:**

A 67-year-old Japanese man with a diagnosis of distal cholangiocarcinoma was referred to our hospital. Thickening of the distal bile duct and an air-space pattern in the upper lobe of the left lung were detected by preoperative computed tomography. He underwent pancreaticoduodenectomy for the distal cholangiocarcinoma. Follow-up chest computed tomography demonstrated that the air-space pattern in the left lung had gradually enlarged. Thoracoscopic left S6 segmentectomy with lymph node dissection was performed 3.5 years after the initial surgery. Histopathology of the resected specimen revealed a solitary metastasis from distal cholangiocarcinoma with lepidic growth. We diagnosed the patient with a solitary synchronous pulmonary metastasis from distal cholangiocarcinoma.

**Conclusions:**

Surgical resection might offer better long-term survival to patients with synchronous pulmonary metastasis from distant cholangiocarcinoma than nonsurgical treatments. Pulmonary metastasis from distal cholangiocarcinoma may exhibit a lepidic pattern. Therefore, because of the possibility of synchronous pulmonary metastasis, pulmonary resection should be considered for patients with lepidic lesions who have been diagnosed with distal cholangiocarcinoma.

## Background

Surgical resection is necessary for curative treatment of distal cholangiocarcinoma. Although systemic chemotherapy is applied to distal cholangiocarcinoma with factors of unresectability such as metastasis to the liver, lung, peritoneum, and distant lymph nodes (LNs), its clinical value remains unsatisfactory [1, 2]. A few recent reports have described the effectiveness of resection for treatment of recurrent cholangiocarcinoma [3–6]. However, none have described resection of synchronous pulmonary metastasis from distal cholangiocarcinoma. We report the first case, to the best of our knowledge, of a slow-growing synchronous pulmonary metastasis from distal cholangiocarcinoma resected 3.5 years after the initial surgery.

## Case presentation

**Fig. 1:**
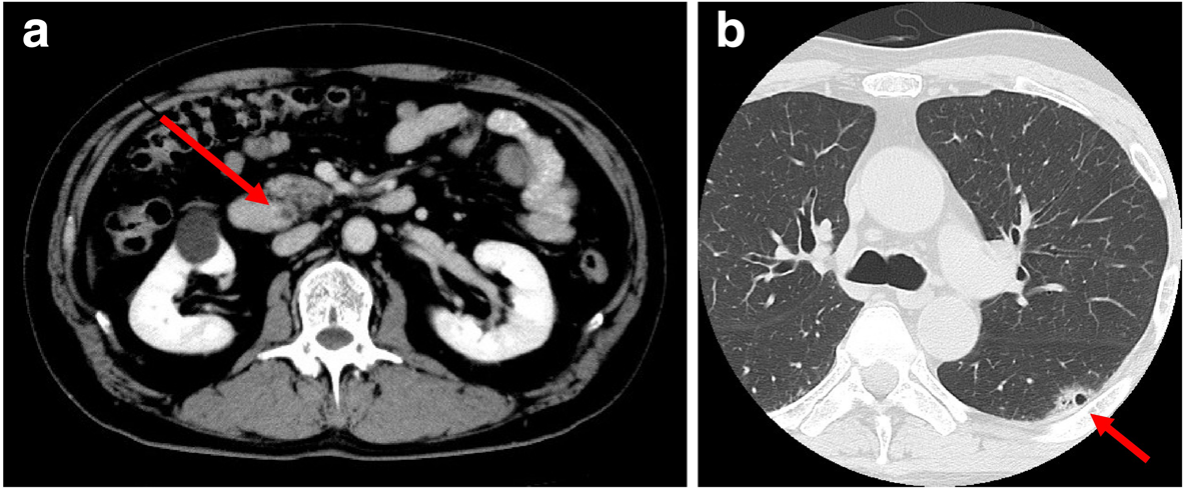
Computed tomography findings. **a** Abdominal computed tomography showing thickening of the distal bile duct (*arrow*). **b** Chest computed tomography showing an air-space consolidation in the upper lobe of left lung (*arrow*)

A 67-year-old Japanese man who had been diagnosed with distal cholangiocarcinoma at a previous hospital was referred to our hospital for surgical treatment. He was a current smoker. His medical and family histories were unremarkable. He had undergone percutaneous transhepatic biliary drainage at a previous hospital because of obstructive jaundice without biliary tract stones. Abdominal computed tomography (CT) revealed thickening of the distal bile duct (Fig. 1a), and chest CT showed an air-space pattern in the upper lobe of the left lung (Fig. 1b). The patient’s serum concentration of carcinoembryonic antigen was elevated at 15.1 ng/ml, and that of carbohydrate antigen was within the reference range.

**Fig. 2:**
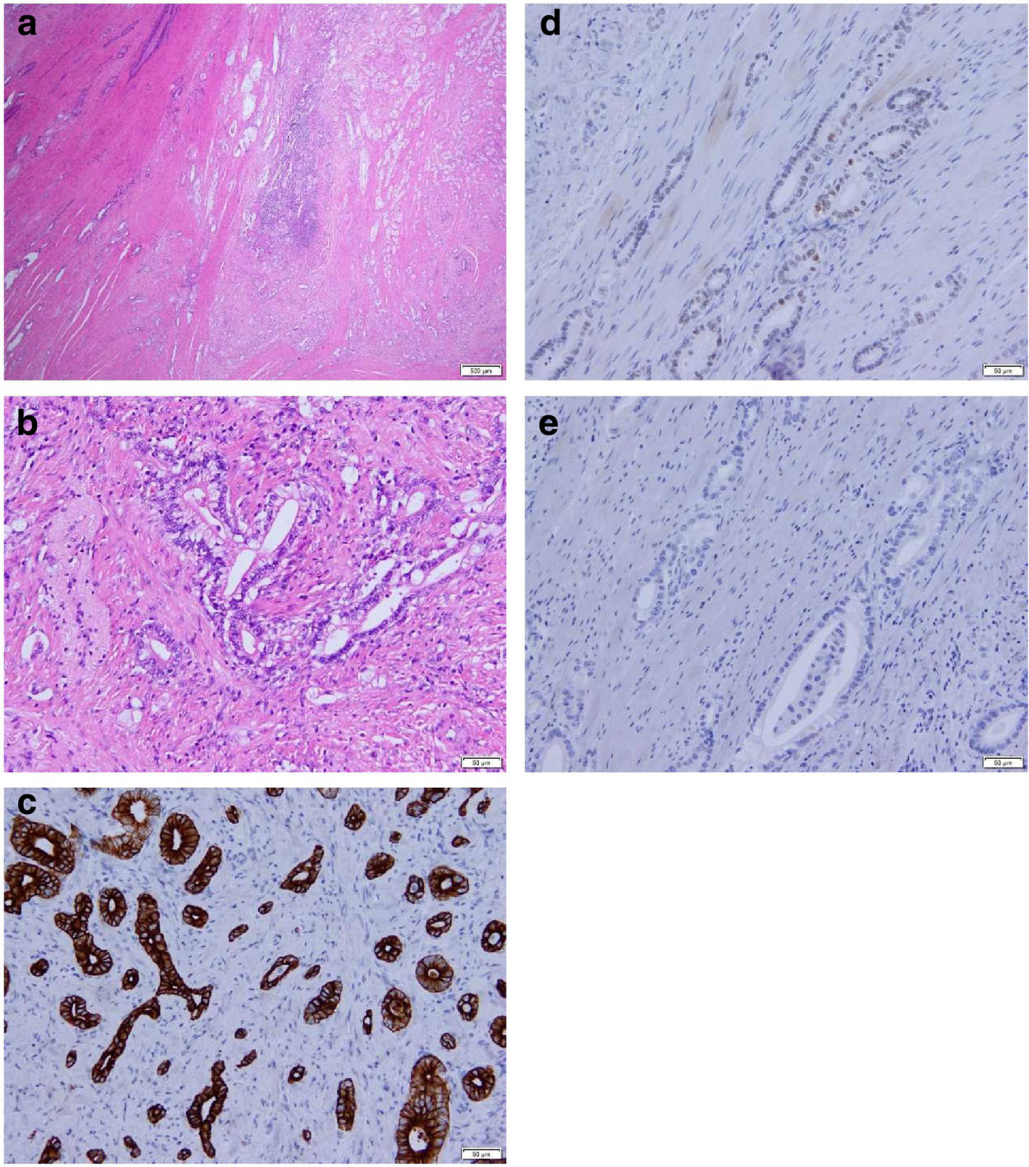
Hematoxylin and eosin (H&E) staining and immunohistochemistry of the resected specimen after pancreaticoduodenectomy. H&E stains show well-differentiated to moderately differentiated adenocarcinoma (**a**, original magnification × 10; **b**, original magnification × 200). Immunohistochemical stains show that the tumor cells were positive for cytokeratin 7 (**c**, original magnification × 200) and CDX-2 (**d**, original magnification × 200) and negative for cytokeratin 20 (**e**, original magnification × 200)

The patient underwent pancreaticoduodenectomy (PD) for the distal cholangiocarcinoma. Because of its small size, the lesion in the left lung could not be diagnosed accurately in terms of whether it was malignant and had the possibility of being pneumonia. Therefore, the lesion was observed. Histological examination of the resected specimen revealed well-differentiated adenocarcinoma invading the pancreas with five regional LN metastases (Fig. 2a, b). Immunohistochemical examination showed that the tumor cells were positive for cytokeratin 7 (CK7) (Fig. 2c) and CDX-2 (Fig. 2d) and negative for CK20 (Fig. 2e). According to the seventh edition of the TNM staging system of the Union for International Cancer Control, the patient’s disease was diagnosed as distal cholangiocarcinoma, T3N1M0, stage IIB. The patient received postoperative adjuvant chemotherapy with the oral fluoropyrimidine S-1 (120 mg/body) for 6 months. S-1 was given for 4 weeks, followed by a 2-week rest. This administration of S-1 was repeated every 6 weeks for up to four cycles.

**Fig. 3:**
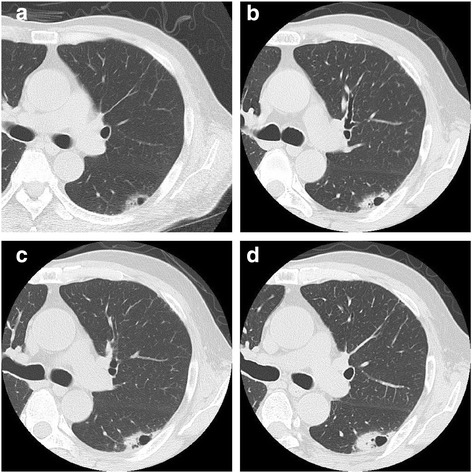
Change in the air-space consolidation in the upper lobe of the left lung after pancreaticoduodenectomy: (**a**) 1 year, (**b**) 2 years, (**c**) 3 years, and (**d**) 3.5 years

**Fig. 4:**
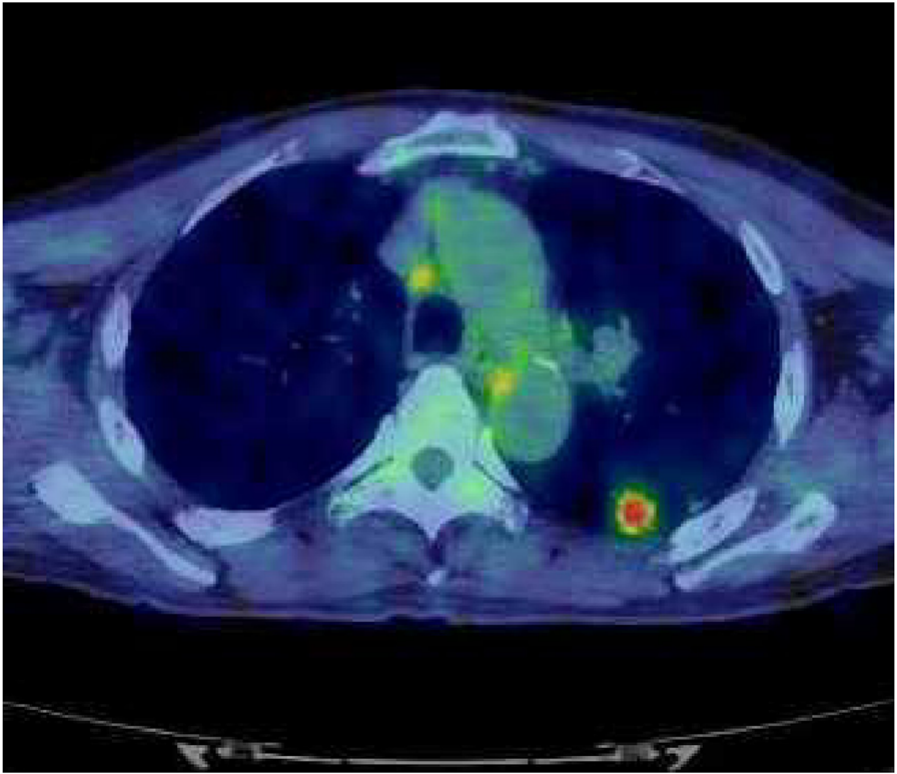
^18^F-fluorodeoxyglucose computed tomography revealing abnormal uptake in the lesion of the left lung with a maximum standardized uptake value of 4.30

Follow-up chest CT was periodically performed about every 4 months during the first 5 years after the initial surgery. Follow-up chest CT demonstrated that the air-space pattern in the upper lobe of the left lung had gradually enlarged to 36.8 mm in diameter with no additional lesions for 3.5 years after PD (Fig. 3a–d). The patient was asymptomatic, and a physical examination including assessment of respiratory function revealed no remarkable findings. The patient’s serum concentration of carcinoembryonic antigen was slightly elevated at 5.6 ng/ml. Biochemical parameters except for carcinoembryonic antigen were within the reference ranges. Positron emission tomography with ^18^F-fluorodeoxyglucose CT detected abnormal uptake in the left lung lesion with a maximum standardized uptake value of 4.30 (Fig. 4). A transbronchial lung biopsy showed malignant findings. However, the definitive diagnosis could not be established.

**Fig. 5:**
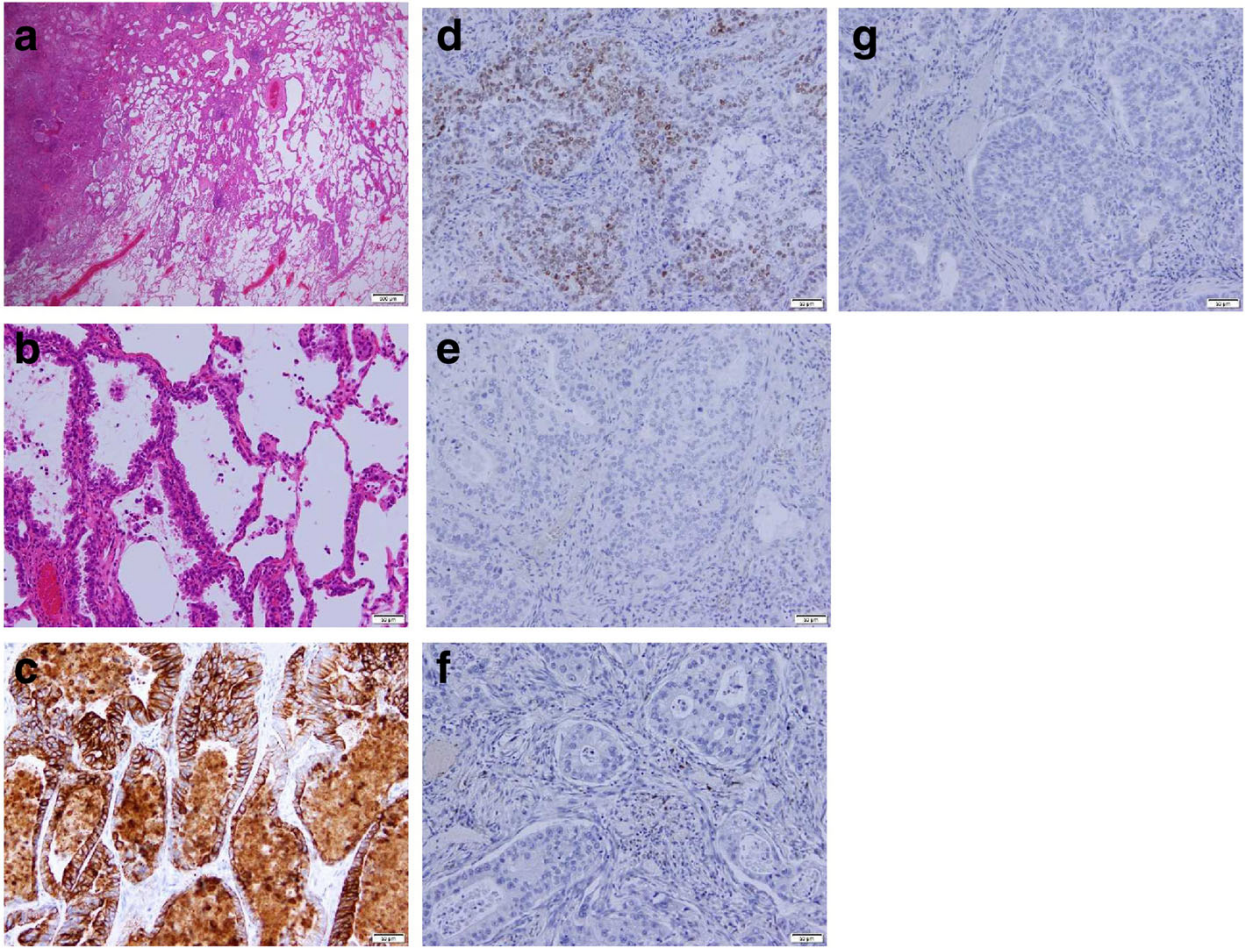
The resected lung specimen resembled the previous distal cholangiocarcinoma. Histopathological examination (H&E staining) of the lung tumor showed tumor cells forming irregular tubular structures with a lepidic pattern (**a**, original magnification × 10; **b**, original magnification × 200). Immunohistochemically, the tumor cells were positive for cytokeratin 7 (**c**, original magnification × 200) and CDX-2 (**d**, original magnification × 200) and negative for thyroid transcription factor 1 (**e**, original magnification × 200), napsin A (**f**, original magnification × 200), and cytokeratin 20 (**g**, original magnification × 200)

With differential diagnoses of either primary lung cancer or solitary pulmonary metastasis from distal cholangiocarcinoma, thoracoscopic left S6 segmentectomy with LN dissection was performed. Histologically, the resected lung specimen showed adenocarcinoma tissue characterized by the formation of irregular tubular structures with lepidic growth (Fig. 5a, b). Immunohistochemical examination revealed that the tumor cells were positive for CK7 and CDX-2 (Fig. 5c, d), similar to the cholangiocarcinoma, and negative for thyroid transcription factor-1, napsin A, and CK20 (Fig. 5e–g). Additionally, an LN metastasis was detected in a resected regional LN. On the basis of these findings, we finally diagnosed the patient with a solitary synchronous pulmonary metastasis from distal cholangiocarcinoma.

## Discussion

To the best of our knowledge, this is the first report of resection of a solitary synchronous pulmonary metastasis with lepidic growth from distal cholangiocarcinoma 3.5 years after PD. This patient’s course suggests two important clinical issues. First, surgical resection of a synchronous pulmonary metastasis from distal cholangiocarcinoma might offer better long-term survival than nonsurgical treatments. Although surgical resection is the only curative treatment for distal cholangiocarcinoma, more than half of patients who undergo curative resection develop recurrences [7], and the median survival time is < 12 months [1, 2]. The lung is the fourth most common site of recurrence after resection of cholangiocarcinoma [5]. In general, systemic chemotherapy or radiation therapy are considered for patients with recurrence, including recurrence of pulmonary metastases from cholangiocarcinoma. However, the clinical outcomes are not satisfactory [1, 2]. A few recent reports have described the effectiveness of resection for treatment of recurrent cholangiocarcinoma in select patients [3–6]. One study on metachronous pulmonary metastasectomy from cholangiocarcinoma revealed that surgery for pulmonary metastasis should be considered in patients with a longer initial disease-free interval [5]. In our patient, we surgically resected a slow-growing lesion in the upper lobe of the left lung with no additional lesions 3.5 years after the initial surgery because the definitive diagnosis could not be established (primary lung cancer versus solitary pulmonary metastasis from distal cholangiocarcinoma). The lesion in the left lung was histopathologically diagnosed as a solitary synchronous pulmonary metastasis. This case indicates that patients with synchronous pulmonary metastasis may have long survival and that pulmonary resection is possible in these patients.

Second, pulmonary metastasis from distal cholangiocarcinoma may exhibit lepidic growth, making it difficult to distinguish from primary lung cancer. In general, pulmonary metastasis is typically described as one or multiple nodules on radiological imaging. However, metastatic pulmonary adenocarcinoma rarely shows lepidic growth on radiological or histopathological examination [8, 9]. Chest CT of pulmonary metastasis from the pancreas, colon, and stomach shows lepidic growth associated with air-space patterns, such as air bronchograms, ground-glass opacities, and consolidation [9]. Additionally, lepidic pulmonary metastasis from perihilar cholangiocarcinoma was reported in an autopsy case [10]. In our patient, preoperative chest CT before PD showed an air-space pattern in the upper lobe of the left lung. The lesion in the lung was diagnosed as pulmonary metastasis with a lepidic pattern from distal cholangiocarcinoma by immunohistochemical examination of the resected specimen 3.5 years after the initial surgery. A regional LN metastasis of the lung was also detected. Finally, we diagnosed the patient with a solitary synchronous pulmonary metastasis from distal cholangiocarcinoma.

## Conclusions

We report the first case, to the best of our knowledge, of a slow-growing synchronous pulmonary metastasis with lepidic growth from distal cholangiocarcinoma resected 3.5 years after the initial surgery. This case is also extremely rare in that we were able to observe the course of the synchronous pulmonary metastasis for a long period of time. Surgical resection might offer better long-term survival to patients with synchronous pulmonary metastasis from distal cholangiocarcinoma than nonsurgical treatments. Pulmonary metastasis from distal cholangiocarcinoma may exhibit a lepidic pattern, making it difficult to distinguish from primary lung cancer. Therefore, pulmonary resection should be considered for patients with lepidic lesions who have been diagnosed with distal cholangiocarcinoma because of the possibility of synchronous pulmonary metastasis.

## Abbreviations

CK: Cytokeratin
CT: Computed tomography
LN: Lymph node
PD: Pancreaticoduodenectomy
